# Clusters of incompatible genotypes evolve with limited dispersal

**DOI:** 10.3389/fgene.2015.00151

**Published:** 2015-04-22

**Authors:** Erin L. Landguth, Norman A. Johnson, Samuel A. Cushman

**Affiliations:** ^1^Computational Ecology Laboratory, Division of Biological Sciences, University of MontanaMissoula, MT, USA; ^2^Department of Biology, Department of Environmental Conservation, and Graduate Program in Organismic and Evolutionary Biology, University of MassachusettsAmherst, MA, USA; ^3^Rocky Mountain Research Station, United States Forest ServiceFlagstaff, AZ, USA

**Keywords:** CDPOP, Dobzhansky–Muller incompatibilities, individual-based simulations, landscape genetics, movement strategies, speciation

## Abstract

Theoretical and empirical studies have shown heterogeneous selection to be the primary driver for the evolution of reproductively isolated genotypes in the absence of geographic barriers. Here, we ask whether limited dispersal alone can lead to the evolution of reproductively isolated genotypes despite the absence of any geographic barriers or heterogeneous selection. We use a spatially-explicit, individual-based, landscape genetics program to explore the influences of dispersal strategies on reproductive isolation. We simulated genetic structure in a continuously distributed population and across various dispersal strategies (ranging from short- to long-range individual movement), as well as potential mate partners in entire population (ranging from 20 to 5000 individuals). We show that short-range dispersal strategies lead to the evolution of clusters of reproductively isolated genotypes despite the absence of any geographic barriers or heterogeneous selection. Clusters of genotypes that are reproductively isolated from other clusters can persist when migration distances are restricted such that the number of mating partners is below about 350 individuals. We discuss how our findings may be applicable to particular speciation scenarios, as well as the need to investigate the influences of heterogeneous gene flow and spatial selection gradients on the emergence of reproductively isolating genotypes.

## Introduction

Hybrids between species often exhibit reduced viability, lower fertility, and/or phenotypic abnormalities. These deleterious traits in hybrids, which are collectively known as hybrid incompatibility, are a form of postzygotic reproductive isolation, and thus are important to the speciation process (Coyne and Orr, 2004). Dobzhansky (1937) and Muller (1942) presented models arguing that hybrid incompatibility usually evolves due to changes in at least two different genetic loci. Genetic studies strongly support the Dobzhansky–Muller model (see references within Coyne and Orr, 2004; Seehausen et al., 2014). In recent years, a growing number of these hybrid incompatibility genes have been identified (reviewed in Johnson, 2010; Presgraves, 2010).

Hybrid incompatibility can also occur between different populations of the same species (e.g., in flour beetles, Demuth and Wade, 2007, in flies, Lachance and True, 2010; in nematodes, Seidel et al., 2008, 2011). Within-species hybrid incompatibility can arise given synthetic deleterious loci, sets of loci wherein individuals with combinations of alleles at more than one locus have low fitness but where possession of one of those alleles has little or no fitness consequence for the carriers (Phillips and Johnson, 1998). Analytical studies (Phillips and Johnson, 1998; Lachance et al., 2011) showed that these synthetic alleles could reach considerably high frequencies (roughly the quartic root of the mutation rate divided by the selection coefficient) in panmictic populations under mutation-selection balance (see also, Lachance et al., 2011). Indeed, synthetic lethality and sterility has been found at appreciable frequencies in populations of *Drosophila melanogaster* (e.g., Lachance and True, 2010).

Can something like hybrid incompatibility evolve within the same population? This question relates to the debate regarding the feasibility of sympatric speciation. Most of the models of sympatric speciation wherein reproductive isolation arises in the face of moderate or strong gene flow involve the counterbalancing force of relatively strong and heterogeneous natural selection. In these models, selection enables nascent species to evolve genetic differences that are incompatible with the evolved differences in the other nascent species (Gavrilets and Vose, 2007; Gavrilets et al., 2007; Nosil and Feder, 2012).

Recently, Eppstein et al. (2009) showed that limited dispersal with small numbers of mate potentials alone can lead to the evolution of clusters of reproductively isolated genotypes despite the absence of any geographical barriers or heterogeneous selection. Such clusters evolved when several loci were underdominant (heterozygotes less fit than either homozygote). Non-additive fitness effects across loci (epistasis) enhanced the likelihood of clustering.

Here, we extend the work of Eppstein et al. (2009), and show that underdominance is not required for clustering of reproductively isolated genotypes. Fitness is determined by epistatic interactions, in form of the well-known Dobzhansky–Muller model. Unlike past simulation studies, which consider migration of individuals between demes (e.g., Gavrilets and Vose, 2007; Gavrilets et al., 2007), we set this work in a landscape genetics framework in which genetic divergence is controlled by gene flow, genetic drift, mutation, and selection as functions of individual-based movement and spatially-explicit interactions with environment.

## Methods

### Simulation program

We used CDPOP v1.0 (Landguth et al., 2012), a landscape genetics tool for simulating the emergence of spatial genetic structure in populations resulting from specified landscape processes governing organism movement behavior. CDPOP models genetic exchange among spatially located individuals as a function of individual-based movement through mate selection and dispersal, incorporating vital dynamics (birth and death rates) and all the factors that affect the frequency of an allele in a population (mutation, gene flow, genetic drift, and selection). The landscape genetics framework of this program is such that individuals move as a probabilistic function of their environment (e.g., as habitat fragmentation increases, ability to disperse across gaps is reduced). These movement functions are scaled to a user-specified maximum dispersal and mate selection distance. This maximum movement value allows a user to control for short- and long-range movement of an organism by constraining all mate choices and dispersal distances to be within that limit, with probability specified by the user-defined movement function (e.g., inverse-square). The order of simulated events follow mate selection with given movement functions, birth and resulting Mendalian inheritance, mortality of adults, and offspring dispersal with given movement functions.

CDPOP v1.0 incorporates multi-locus selection, which is controlled via spatially-explicit fitness surfaces for each genotype under selection (Wright, 1932; Gavrilets, 2000). For example, in the case of a single two-allele locus, three relative fitness surfaces would be specified for the three genotypes (AA, Aa, and aa) from the two alleles, A and a. Selection is then implemented through differential survival of offspring as a function of the relative fitness of the offspring's genotype at the location on that surface where the dispersing individual settles (Landguth et al., 2012). CDPOP yields genetic patterns consistent with Wright–Fisher expectations when parameterized to match Wright-Fisher assumptions in simulations (Landguth and Cushman, 2010), as well as producing theoretical changes in allele frequency under selection for single and double diallelic locus (Landguth et al., 2012). For more details, see Landguth et al. (2012).

### Simulation scenarios

**Figure 1:**
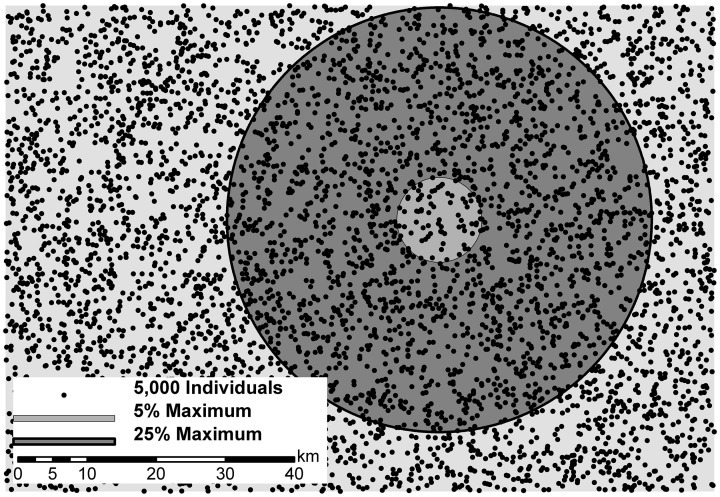
**Five thousand randomly located individuals (dots) on a landscape surface of isolation-by-distance (IBD; light-gray background)**. Two example circles represent the respective dispersal distance/kernel and potential mate pairings for the center individual. Five percent of maximum movement (radius of 6.2 units; medium-gray inner circle) and 25% maximum movement (radius of 30.8 units; dark-gray outer circle) compared to the maximum distance on this landscape of 123.2 units.

We conducted an isolation-by-distance (IBD) simulation modeling experiment across a range of dispersal strategies. We used eight movement distances: 2.5, 5, 7.5, 10, 15, 20, 25, and 100% of the maximum extent of the landscape (Figure 1) with 5000 randomly placed individuals. These dispersal distances correspond to a broad range of possible dispersal destinations for a given offspring, as well as available mating partners for a given individual (ranging from 20 to 5000; Table 1). Mating pairs of individuals and dispersal distance locations of offspring were unbiased for males and females and were chosen based on a weighted random draw from the inverse-square probability function constrained within the specified movement limit. This movement function allowed individuals to most often mate with nearest neighbors, as well as disperse to nearest neighboring locations with occasional long range dispersal within the specified movement limit. Mating parameters were set in CDPOP to represent a population that was dioecious, with both females and males mating with replacement. Reproduction began at birth and the number of offspring produced followed a Poisson process (λ = 4). Thus, a high rate of reproduction maintained a constant population size of 5000 individuals producing an excess number of offspring each generation that were discarded once all 5000 locations were filled through the dispersal process (i.e., forcing individuals out of the simulation study once all available home ranges were occupied, e.g., Balloux, 2001; Landguth and Cushman, 2010).

**Table 1:** **Maximum movement distance area and average number of individuals that occupy the corresponding areas based on a population density for the given simulation landscape of 0.691-unit^2^ (5000 individuals in the total extent area, 7237.60-unit^2^)**.

**Movement distances (%)**	**Average number of individuals in movement distance**	**Average proportion of individuals in movement distance**
100	5000	1.000
25	2058	0.412
20	1317	0.263
15	741	0.148
10	329	0.066
7.5	185	0.037
5.0	82	0.016
2.5	21	0.004

In all scenarios we used CDPOP v1.0 to simulate individual genetic exchange over 1000 non-overlapping generations (100% adult mortality) as a function of individual-based movement, mating, dispersal, and selection. All simulated populations contained 22 diallelic loci (two of which were under selection and 20 of which were assumed to be neutral) with a mutation rate of 0.0005 (near the lower range of mammalian microsatellite mutation rates), free recombination, and no initial linkage disequilibrium. As the program simulates stochastic processes, we ran 10 Monte Carlo replicates for each dispersal distance to quantify the mean and variability of the genetic structure, and tracked the total inbreeding coefficient, *F* (Wright, 1922), for only the 20 neutral loci across generations to show differences in genetic differentiation among scenarios. Because we used a continuously-distributed population with no subpopulations defined, *F* was calculated with the equation 1 – *H_o_*/*H_e_*, where all individuals were pooled to calculate the observed heterozygosity (*H_o_*) and expected heterozygosity (*H_e_*). This equation reflects the Whalund effect as expected from population genetics whenever there is substructure (Allendorf and Luikart, 2007), allowing us to test for spatial genetic structure operating at all movement ranges.

Following the Dobzhansky–Muller model, we considered the two-locus, two-allele selection model. For the Dobzhansky–Muller model, there are many initial starting configurations to consider along with mutation models (see Johnson, 2010 for review). We simulated a system of 5000 individuals (preliminary simulations with 10,000 individuals yielded similar results (unpublished data)) that were initially monomorphic at the first two loci with genotype, AAbb (e.g., nine possible genotypes exist in the two-locus, two-allele selection model, see below). All remaining 20 loci were given an initial uniformly distributed random allele assignment (maximum allelic diversity for the remaining 20 loci, resulting in a mean *H_e_* = 0.45), because no closed form solution for the frequency distribution of alleles of microsatellites exists (Haasl and Payseur, 2010). The first locus under selection was allowed to have forward mutation for A (A to a) and the second locus under selection was allowed backward mutation for b (b to B). The remaining 20 loci followed the *k*-th allele mutation model, a commonly used mutation model (Balloux, 2001; Haasl and Payseur, 2010), which selects another allele at random with the given mutational rate (0.0005 in these simulations). We assumed that alleles a and B are incompatible and individuals that have these two alleles simultaneously have zero viability. This was implemented through relative fitness surfaces of 0.0 across the landscape for the genotypes AaBB, AaBb, aaBB, and aaBb. The other combinations of alleles are compatible, and the corresponding genotypes, AABB, AABb, AAbb, Aabb, and aabb, were given an equivalent fitness of 1.0 across the landscape. In this model, all offspring of matings between individuals AABB and aabb will have heterozygous genotype AaBb and thus, will be inviable or sterile.

### Evaluating clusters of reproductive isolation

In a continuously distributed population, such as simulated in this study, reproductive isolation needs to be carefully defined. An extreme interpretation of reproductive isolation under the Dobzhansky–Muller model is the simultaneous occurrence of two individuals that would produce incompatible hybrids: such as an AABB individual and an aabb individual. One could imagine however, that this single event could occur by chance quite often in a large population, but that the successful propagation of these individuals into spatial clusters (i.e., subpopulations) might occur with a lesser chance. Therefore, we defined the occurrence of reproductive isolation in a continuously distributed population as the combination of two criteria: (1) an occurrence of a spatial cluster of individuals with genotype AABB that emerges simultaneously with another spatial cluster of individuals with genotype aabb (RI event) and (2) a RI event persisting in consecutive generations.

To define an RI event, we used the density-based spatial clustering algorithm (DBSCAN; Ester et al., 1996), which finds spatial clusters if they contain sufficiently many points (*k*) within a neighborhood (ε). From Ester et al. (1996), we used the sorted *k*-dist graph heuristic method and set *k* = 4 to find threshold, *ε* 2000-m (Figure S1). Then, the number of generations at which two separate clusters (AABB and aabb, respectively) emerged with the above criteria (RI events) was reported and averaged across the 10 Monte Carlo runs for each dispersal scenario. To assess persistence of RI events, we simply recorded the duration (in generations) of each RI event and reported the average time duration across each replicate and for each dispersal strategy.

## Results

**Figure 2:**
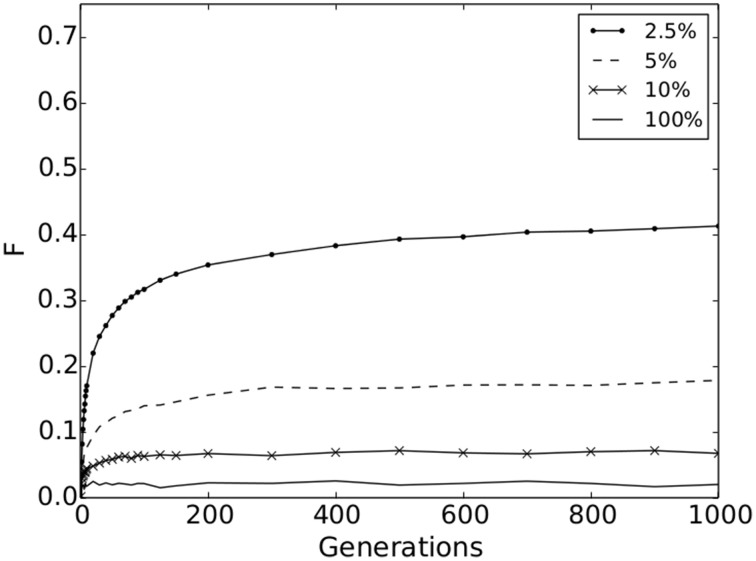
**Population structure**. *F*-values for the simulation scenarios: 2.5, 5, 10, and 100% of the maximum movement distance on the surface (dot-solid line, dashed line, x-solid line, and solid line, respectively). Confidence intervals for the 10 replicates are not visible at this scale.

We found that significant genetic differentiation emerged for all movement strategies, showing that IBD results in spatial genetic structure in all scenarios. However, at short-range movement distances (2.5% maximum dispersal and an average of 21 potential mate partners) genetic differentiation is much stronger with *F* = 0.41 [with a confidence interval (0.412, 0.414)] after 1000 generations compared to a *F* = 0.02 [with a confidence interval (0.020, 0.021)] for the longest-range movement distance (100% maximum dispersal and 5000 potential mate partners; Figure 2). Values for maximum movement strategies above 10% are not shown in Figure 2, but range from *F* = 0.02 to 0.07. This shows that individuals must have limited dispersal ability relative to their landscape extent and few mating options with respect to entire population under IBD in order for large signals in genetic structure to emerge (e.g., Landguth et al., 2010).

**Figure 3:**
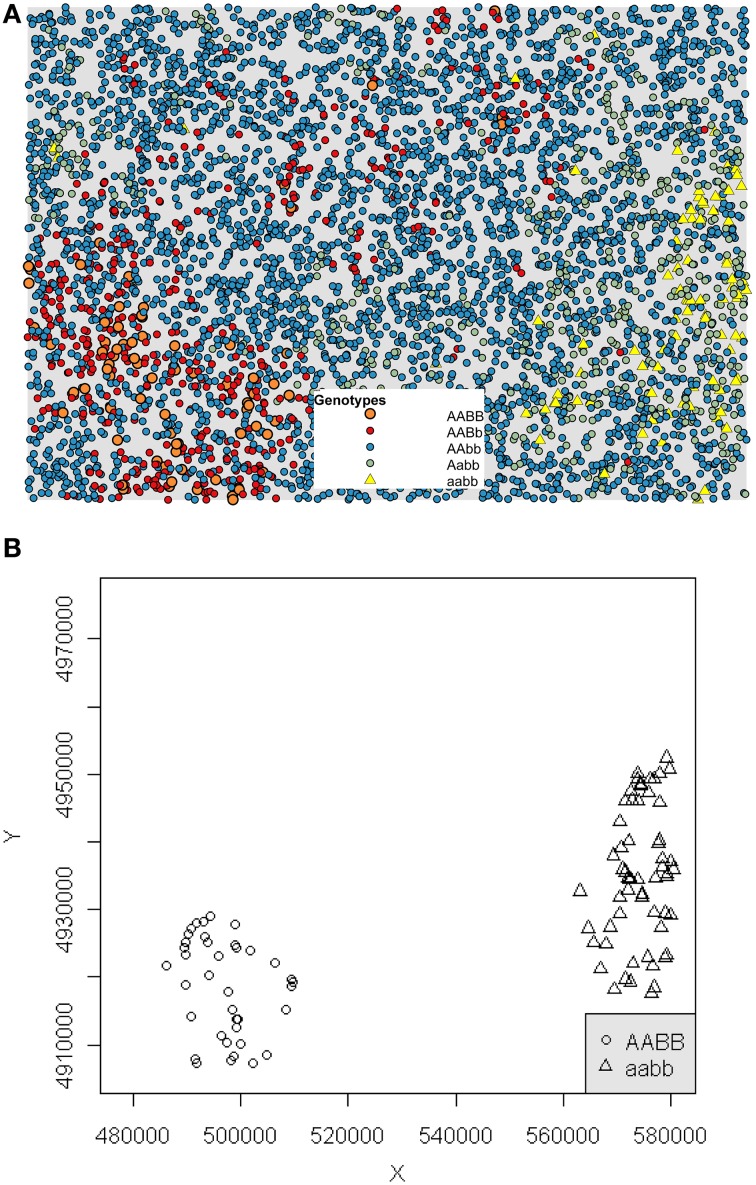
**RI event**. The five genotypes plotted for an example 5% maximum movement distance at generation 900 at which a significant RI event occurred **(A)**. The DBSCAN algorithm identified clustered groups of AABB (circles) and aabb (triangles) in **(B)**.

We detected spatial clustering of incompatible genotypes in the scenarios in which the movement distance is low (less than 10% of the maximum movement and an average of 329 potential mate partners). The highly constrained mating and dispersal movements in these scenarios frequently resulted in occurrences of reproductive isolation. In contrast, no occurrences of reproductive isolation occurred in scenarios with dispersal distances greater than 10% of the maximum extent of available habitat, suggesting a non-linear decrease in reproductive isolation as dispersal ability is increased, as well as number of potential mate partners. A total of 864 significant clustering events occurred within 1000 generations at the 2.5% maximum dispersal distance. In contrast, on average 6.5 reproductively isolated clusters occurred at the 10% dispersal distance (Table 2). Figure 3A shows a significant clustering event for one example generation of the 5% maximum movement with the DBSCAN algorithm separating out the hybrid incompatible genotypes in Figure 3B.

**Table 2:** **Mean number of significant occurrences of a RI Event; Max and mean duration of an RI Event**.

	**2.5%**	**5%**	**7.5%**	**10%**	**15%**	**20%**	**25%**	**100%**
**RI EVENT**								
**Mean**	863.5	177.0	49.3	6.5	0	0	0	0
**95% CI**	840.12–886.88	158.87–195.13	40.70–57.90	4.05–8.95	NA	NA	NA	NA
**RI EVENT DURATION**								
**Max**	494	70	14	2	NA	NA	NA	NA
**Mean**	16.6	2.1	1.5	1.1	NA	NA	NA	NA

*All values are in units of generation*.

We measured the duration for each spatial clustering event of incompatible genotypes across the replicate simulations (Table 2). The mean duration for the 2.5% dispersal strategy was 16.6 generations, with a maximum duration of 494 generations. This is compared to a single maximum time duration event occurring in the 10% dispersal strategy of two generations and no other events greater than a single generation resulting in a mean of 1.1 generations. This suggests that persistent reproductive isolation only occurs when dispersal ability is very limited, resulting in very small genetic neighborhoods (e.g., less than 10% of the extent of available habitat and potential mate partners is approximately less than 7% of the total population).

## Discussion

Our results suggest that under strong selection clusters of incompatible genotypes will readily evolve within continuously distributed populations when dispersal distances and potential mating choices are small relative to entire landscape extents and population size, respectively. In these simulations, this phenomenon is weak when movement distances occur at a range up to 10% of the maximum landscape space, but becomes increasingly strong as migration is further reduced. In combination with short-range dispersal distances, limited mating distances, and the resulting pattern of few potential available mates, also increase the emergence of RI events. Short mating distances reduce the rate at which genes moved through the population and reduce local effective population sizes such that local genetic structure would be maintained and not swamped by the homogenizing effects of high rates of gene flow. When mating and dispersal are very limited, reproductive isolation frequently evolves and reproductively isolated clusters may be highly persistent over time. How long reproductively isolated clusters persist in nature is unknown, but we show a maximum duration event occurring for 494 generations. Eppstein et al. (2009) previously had found hybrid incompatibility would evolve under restricted migration and multilocus underdominance. The extent to which multilocus underdominance occurs in nature is also not known, however. Our study, which does not involve underdominance, extends the range of genetic conditions under which reproductive isolation can arise by restricted migration.

Hybrid incompatibility is expected to evolve under a number of circumstances. In addition to allopatry (e.g., Mayr, 1963; Coyne and Orr, 2004; Gavrilets, 2004), other scenarios include various modes of diversifying selection (Doebeli and Dieckmann, 2003; Schluter, 2009; also see Savolainen et al., 2006 for a case example), and temporally heterogeneous selection (Johnson and Porter, 2000; Porter and Johnson, 2002). Here, like Eppstein et al. (2009), we have invoked none of the above scenarios, and yet, reproductive isolation still evolves under strong selection and restricted migration. Thus, restricted migration should be added to the list of circumstances that potentially promote the evolution of hybrid incompatibility, and hence, speciation. We also argue that our results establish a null model for examining the evolution of hybrid incompatibility in populations of organisms with limited dispersal; the presence of hybrid incompatibility in such cases implies neither geographic barriers nor heterogeneous selection.

The incompatible genotypes we found resemble synthetic deleterious loci (Dobzhansky, 1937; Phillips and Johnson, 1998) in some respects, but are distinct. Analytical studies (Phillips and Johnson, 1998; Lachance et al., 2011) showed that these synthetic alleles could reach considerably high frequencies (roughly the quartic root of the mutation rate divided by the selection coefficient) in panmictic populations under mutation-selection balance (see also, Lachance et al., 2011). Indeed, synthetic lethality and sterility has been found at appreciable frequencies in populations of *Drosophila melanogaster* (e.g., Lachance and True, 2010). Synthetic deleterious loci and the reproductive isolation seen in this study both involve multilocus incompatibilities, but there are differences. The population genetic theory of synthetic deleterious loci predicts heterozygous carriers to be at appreciable frequencies (Phillips and Johnson, 1998; Lachance et al., 2011), but does not predict the clusters of reproductively isolated genotypes that we observe in this study. In addition, these past simulation studies assumed panmixia and infinite population sizes (Phillips and Johnson, 1998; Lachance et al., 2011). In contrast, the simulations here involve finite population size and individual-based, spatially-explicit movement with limited migration.

Restricted migration and hybrid incompatibility may act in a positive feedback loop. In the reinforcement model, secondary contact between nascent species with some degree of hybrid incompatibility may lead to the evolution of premating isolating barriers (Fisher, 1930; Dobzhansky, 1940; Servedio and Noor, 2003). Another possible outcome of such secondary contact is restricted migration (Fisher, 1930). If dispersal behavior is at least partially heritable and heterotypic matings are costly, then those individuals that reduce movement would have a selective advantage. Analytical models demonstrate that reduced migration is a feasible consequence of secondary contact under certain conditions (Yukilevich and True, 2006).

Under what conditions would we expect limited dispersal to be a primary driver of the evolution of incompatibility in the absence of any barrier? This scenario would be likely in organisms that engage in limited individual dispersal due to physiological or behavioral reasons. One example may be the famed *Ensatina* salamander ring species complex. As in many plethodontid salamanders, migration is restricted in these *Ensatina* species (Wake, 1997; Kuchta et al., 2009). This scenario would also be favored when the number of possible Dobzhansky–Muller incompatibilities in the genome is high. Limited dispersal would be less likely to be the major driver of the evolution of hybrid incompatibility when other modes such as divergent selection (Schluter, 2009; Nosil and Feder, 2012) or genomic conflict (Johnson, 2010; Presgraves, 2010) are operating.

Our results suggest that hybrid incompatibility should be relatively easy to evolve in relatively short periods of time in species with limited dispersal. So, why don't we see more hybrid incompatibility in natural populations? One possibility is that hybrid incompatibility is more common than we expect, but that it has not been sufficiently sampled. Studies finding hybrid incompatibility within putative species support this hypothesis (e.g., Edmands, 2002; Demuth and Wade, 2007). Another possibility is that in most species the number of mutations at loci that act in Dobzhansky–Muller incompatibilities is limited.

This work extends our understanding of the evolution of clusters of reproductively isolated genotypes by putting it in an explicit landscape genetics context. Most prior studies of the geographic context of the evolution of reproductive isolation have been cast in the framework of discrete populations linked by dispersal. In contrast, our analysis investigates emergence of reproductively isolated clusters within a continuously distributed population governed by isolation by distance. Studies of continuously distributed populations and spatial processes, such as isolation by distance and isolation by resistance form the foundation of the emerging field of landscape genetics. Framing important evolutionary questions, such as the origin of reproductive isolation, in a spatially-explicit landscape genetics framework provides an important first step in exploring a wide range of the potential effects of spatial dependence in evolution.

Although this is the first study to address spatially-explicit individuals moving as probabilistic functions of their landscape, it is still a simple isolation-by-distance model. Future research should address how landscape heterogeneity affects the generation of cluster of reproductively isolated genotypes through landscape restricted migration. For example, how does population subdivision into separate patches and spatially heterogeneous patterns of resistance to dispersal affect the emergence of reproductively isolating clusters? How do spatial selection gradients influence the emergence of reproductively isolating clusters? Furthermore, how do changing landscapes influence transient vs. equilibrium dynamics and thus, change the rate of the emergence of reproductive isolation? These questions have direct relevance for mosaic hybrid zones (e.g., Ross and Harrison, 2002) and how they are shaped by individual-based movement strategies, heterogeneous and changing landscapes, spatial selection gradients, and their interactions. Future simulations should combine a range of landscape complexities (affecting dispersal) in combination with spatial selection gradients within this individual-based landscape genetics framework to elaborate on the processes controlling the emergence of reproductive isolation. In addition, future studies should evaluate how changing the dominance and strength of the incompatibilities, as well as recombination and mutational models influences the outcome.

### Conflict of interest statement

The authors declare that the research was conducted in the absence of any commercial or financial relationships that could be construed as a potential conflict of interest.

## Supplementary material

Figure S1 **Sorted 4-dist graph used to find DBSCAN parameter, ε**. The location of the first inflection point corresponds to approximately ε = 2000. To define an RI event, we used the density-based spatial clustering algorithm (DBSCAN; Ester et al., 1996), which finds spatial clusters if they contain sufficiently many points (*k*) within an ε-neighborhood (ε). From Ester et al. (1996) we used the sorted *k*-dist graph heuristic method and set *k* = 4 to find threshold, ε. This method is based on the observation that *k*-nearest neighbor distance can find an indication of the distribution of closeness of data, i.e., an ε for which a minimum number individuals in the population can form spatial clusters. For a given *k* = 4, we define a function 4-dist, mapping each point to the distance from its fourth-nearest neighbor. When we sorted the 4-dist values in descending order, we estimated a threshold value of ε = 2000 m (Figure S1; Ester et al. showed that a *k* > 4 did not significantly differ from a 4-dist graph). Then, the generations at which two separate clusters (AABB and aabb, respectively) emerge with the above criteria across the six simulation scenarios was reported and averaged across the 10 Monte Carlo runs.
