# Supplementary figures and images for: Clusters of incompatible genotypes evolve with limited dispersal
Source: Front Genet. 2015 Apr 22;6:151. doi: 10.3389/fgene.2015.00151 (PMC4406094; doi:10.3389/fgene.2015.00151)

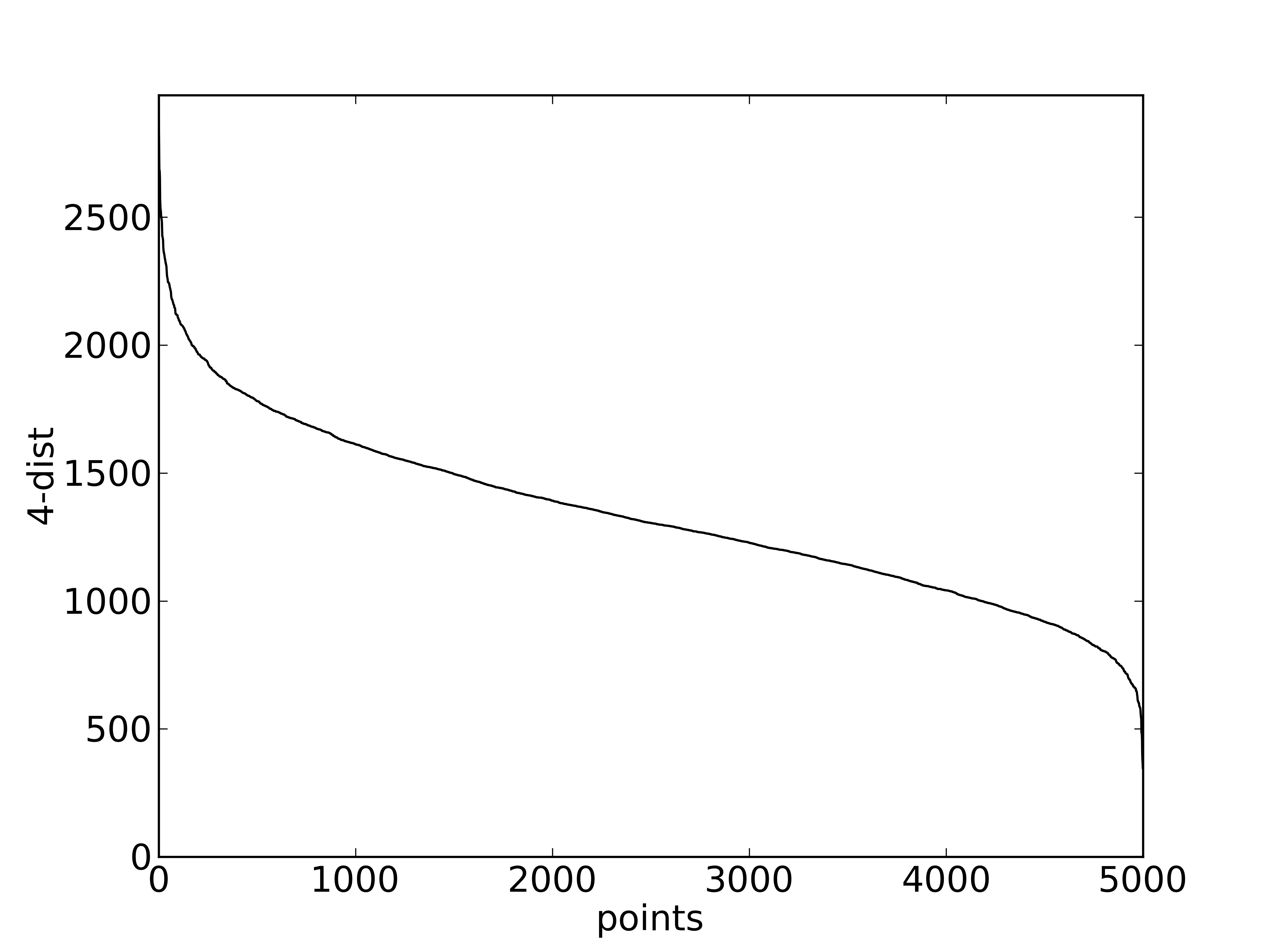

Supplement: Figure S1 — Sorted 4-dist graph used to find DBSCAN parameter, ε. The location of the first inflection point corresponds to approximately ε = 2000. To define an RI event, we used the density-based spatial clustering algorithm (DBSCAN; Ester et al., 1996), which finds spatial clusters if they contain sufficiently many points (k) within an ε-neighborhood (ε). From Ester et al. (1996) we used the sorted k-dist graph heuristic method and set k = 4 to find threshold, ε. This method is based on the observation that k-nearest neighbor distance can find an indication of the distribution of closeness of data, i.e., an ε for which a minimum number individuals in the population can form spatial clusters. For a given k = 4, we define a function 4-dist, mapping each point to the distance from its fourth-nearest neighbor. When we sorted the 4-dist values in descending order, we estimated a threshold value of ε = 2000 m (Figure S1; Ester et al. showed that a k > 4 did not significantly differ from a 4-dist graph). Then, the generations at which two separate clusters (AABB and aabb, respectively) emerge with the above criteria across the six simulation scenarios was reported and averaged across the 10 Monte Carlo runs. [file Image1.PNG]
